# A Simple Geotracer Compositional Correlation Analysis Reveals Oil Charge and Migration Pathways

**DOI:** 10.1038/srep23066

**Published:** 2016-03-11

**Authors:** Yunlai Yang, Khaled Arouri

**Affiliations:** 1 EXPEC Advanced Research Center, Saudi Aramco, Dhahran 31311, Kingdom of Saudi Arabia

## Abstract

A novel approach, based on geotracer compositional correlation analysis is reported, which reveals the oil charge sequence and migration pathways for five oil fields in Saudi Arabia. The geotracers utilised are carbazoles, a family of neutral pyrrolic nitrogen compounds known to occur naturally in crude oils. The approach is based on the concept that closely related fields, with respect to filling sequence, will show a higher carbazole compositional correlation, than those fields that are less related. That is, carbazole compositional correlation coefficients can quantify the charge and filling relationships among different fields. Consequently, oil migration pathways can be defined based on the established filling relationships. The compositional correlation coefficients of isomers of C_1_ and C_2_ carbazoles, and benzo[a]carbazole for all different combination pairs of the five fields were found to vary extremely widely (0.28 to 0.94). A wide range of compositional correlation coefficients allows adequate differentiation of separate filling relationships. Based on the established filling relationships, three distinct migration pathways were inferred, with each apparently being charged from a different part of a common source kitchen. The recognition of these charge and migration pathways will greatly aid the search for new accumulations.

Improved understanding of the migration pathways and distances travelled by crude oils from source rocks to reservoirs is vital to aid the search for new petroleum accumulations. Such information is not provided explicitly by seismic or other remote-sensing surveys. Geochemists have, therefore, sought to utilise molecular markers, commonly referred to as geotracers, to help identify the absolute or relative distances that crude oils have migrated^1^,^2^. One group of compounds that have been recognised as useful in this respect are carbazole and its derivatives, which occur naturally in crude oils (Fig. 1). Carbazoles are aromatic heterocyclic organic compounds, the core of which is based on a tricyclic structure, consisting of two six-membered benzene rings fused on either side of a five-membered nitrogen-containing ring. Previous studies have reported the use of carbazoles to estimate oil migration distances, based on evidence showing that, during oil migration, the concentration of carbazoles decreases and isomer ratios change, due to preferential removal from the migrating oils by adsorption onto mineral surfaces and/or partitioning into pore waters^2^,^3^,^4^,^5^,^6^,^7^,^8^,^9^,^10^,^11^,^12^,^13^,^14^.

Naturally occurring geotracers (such as carbazoles) are usually measured in reservoired oils. The concentration of carbazoles in oils represents the difference between the initial concentration expelled from the source rock and the amount removed during oil migration and accumulation^11^. Initial concentrations of carbazoles depend on both source rock type and maturity^2^,^12^,^15^,^16^,^17^,^18^,^19^,^20^,^21^,^22^,^23^. Loss of carbazoles during migration and accumulation processes is complicated and depends on a variety of factors including migration channel size, host rock porosity, specific surface area and adsorption capacity, volume of reservoired oil and relative migration distance^11^. Since the concentration of carbazoles can be affected by the significant factors outlined above, it is very difficult to estimate oil migration distances directly from carbazole concentrations.

We report a novel method for locating oil charge and migration pathways using carbazole compositional correlations between different fields. Five oil fields, located in the Summan exploration area in the north-eastern part of Saudi Arabia (Fig. 2), were utilised as a case study to test the proposed methodology and illustrate the general procedure for assessing oil migration pathways.

## Results

**Figure 2:**
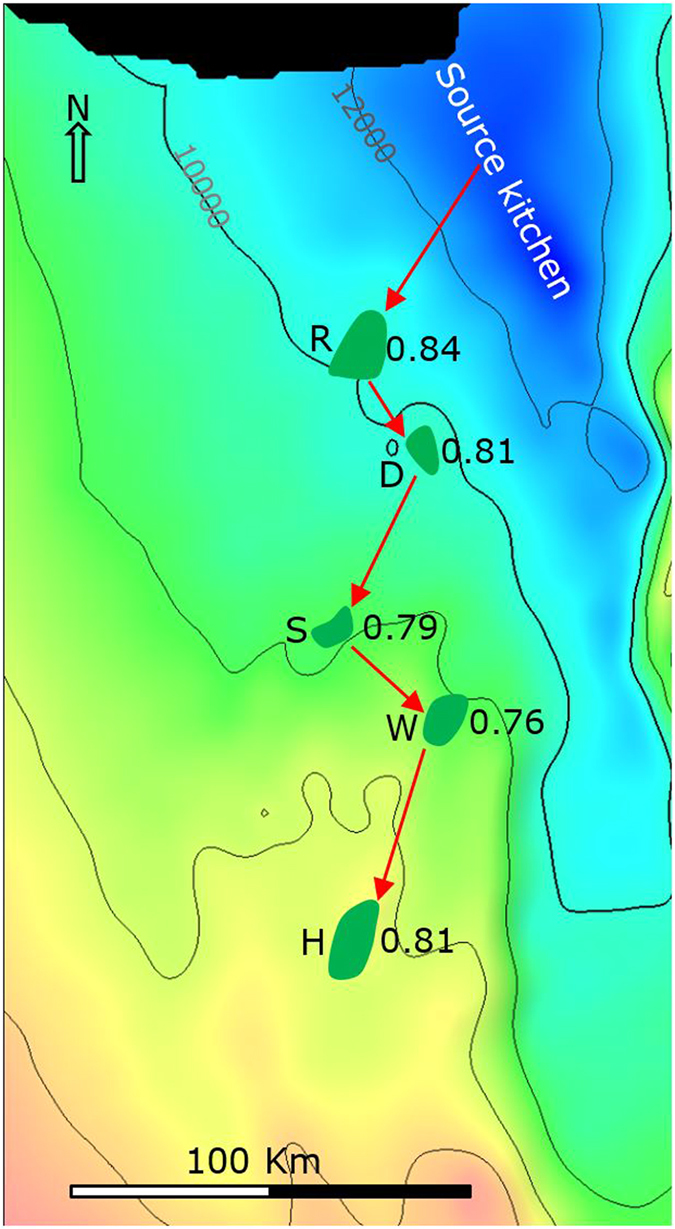
Location of the five oil fields and the projected location of the correspondent source kitchen. The location and size of the fields are from Al-Husseini (1997, courtesy of GeoArabia)^29^. The surface is top of the Arab Formation of the Late Jurassic Epoch, prepared from seismic survey data (source: Saudi Aramco). The contours are depth below mean sea level in feet. Source kitchen is in the Hanifa and Tuwaiq Mountain formations of the Middle Jurassic Epoch^24^. Red arrows indicate one previous assumption of migration pathway: source kitchen → R → D → S → W → H. The number on the right of each field is equivalent vitrinite refelctance (%) calculated from methylphenanthrene index^26^.

**Figure 3:**
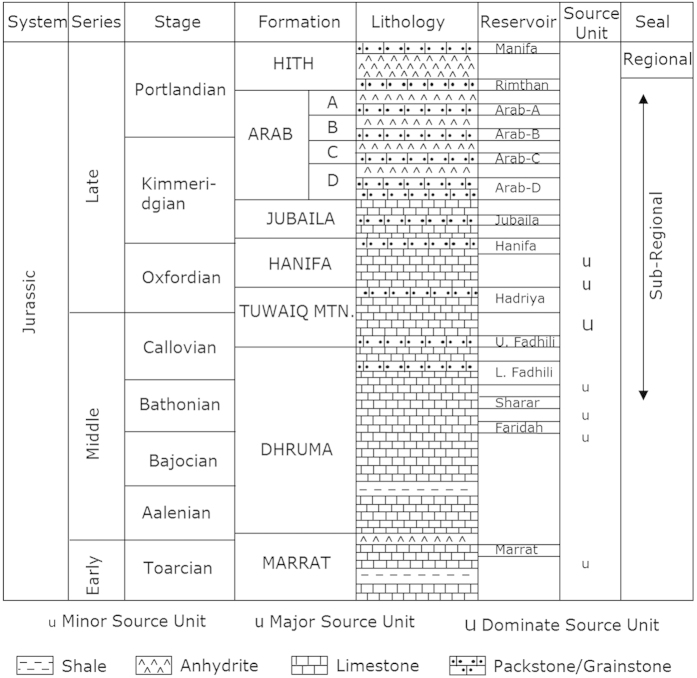
Generalized stratigraphic column for Jurassic sediments showing ages, formation, lithology, reservoirs and source rocks for the key components of the Jurassic petroleum system in Eastern Saudi Arabia (modified from Cole *et al*., 1994)^24^.

Figure 2 shows the locations of the five oil fields and the source kitchen (Gotnia Basin). There are four limestone reservoirs in the fields, namely Arab-A, -B, -C and -D of the Late Jurassic age, in each case separated and capped by anhydrite^24^ (Fig. 3). Oils from these fields were generated from source rocks of the Hanifa and Tuwaiq Mountain formations of the Middle – Late Jurassic^24^ (Fig. 3). The reservoirs of the five oil fields are 5,000–10,000 ft shallower than the source kitchen (reference to Figs 2 and 3). One assumption in the company prior to our study is that these oils were generated in the deep source kitchen located in the north and migrated tens to hundreds of kilometres to the south, filling the five fields sequentially (source kitchen → R → D → S → W → H, Fig. 2).

Samples were chosen solely from the Arab-A reservoir in each field from Modular formation Dynamics Tester (MDT), in order to avoid uncertainties arising from the use of samples from different reservoir units. All the fields discussed herein were discovered between 1974 and 1981; analysis was restricted by the number of oil samples that were properly documented, available and believed to be in good storing conditions prior to analysis. As such, each of the five fields is represented here by one oil sample.

**Figure 4:**
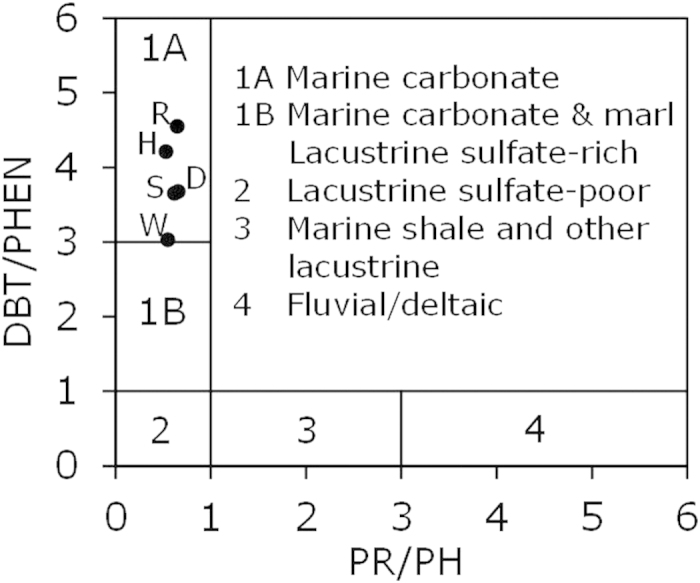
Depositional environments and lithologies of source rock of the five crude oils inferred from cross-plot of dibenzothiophene/phenanthrene (DBT/PHEN) vs. pristane/phytane (PR/PH) (from Hughes *et al*., 1995)^25^.

Source types and maturity of the five oils were inferred from saturated and aromatic hydrocarbon parameters, since the source rock samples were not available. Dibenzothiophene/phenanthrene versus pristane/phytane plot^25^ suggest a common carbonate source rock (Fig. 4). Methylphenanthrene indices^26^ indicate a narrow maturity range of 0.76–0.84% vitrinite reflectance equivalent (Fig. 2).

**Figure 5:**
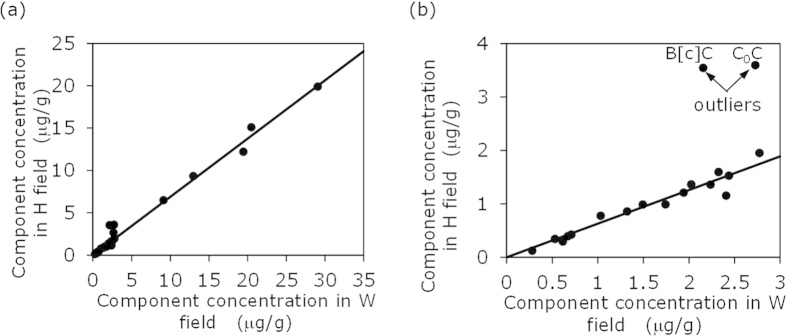
Compositional correlation between oils from the fields W and H. (**a**) All components. (**b**) Low-concentration components (C_0_ carbazole, isomers of C_1_ and C_2_ carbazoles, benzo[a]carbazole and benzo[c]carbazole). The measured values of C_0_ carbazole (C_0_C) and benzo[c]carbazole (B[c]C) from the field H, marked, are considered outliers. In the diagrams, each data point represents a different carbazole component.

Concentrations of various carbazole components determined in the oils are summarised in Table 1. For convenience, concentrations of carbazole components are termed carbazole compositional concentrations hereinafter. When carbazole compositional concentrations of the sample from field H were compared with those of the sample from the nearest field W (Fig. 5), it was found that data points of C_0_ carbazole and benzo[c]carbazole are outliers (marked in Fig. 5b). Subsequent examination of cross plots of the measured compositional concentrations for all combinational pairs of the five fields revealed that the measured concentrations of C_0_ carbazole and benzo[c]carbazole of the sample from field H are outliers (highlighted in Table 1). As a result, the measured concentrations of C_0_ carbazole and benzo[c]carbazole were excluded from the subsequent compositional correlation analysis.

**Figure 6:**
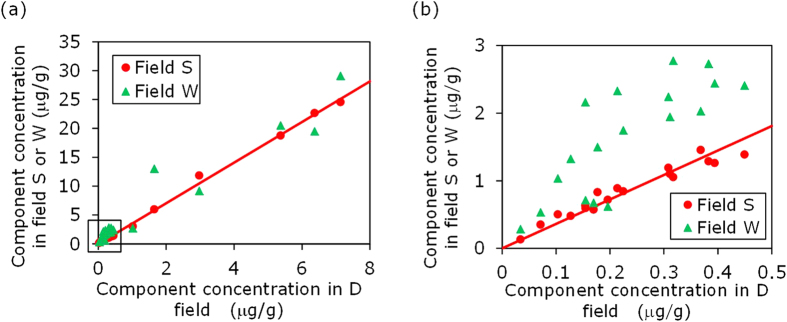
Carbazole compositional correlation between oils from fields D, S and W. (**a**) All components. (**b**) Low-concentration components (C_0_ carbazole, isomers of C_1_ and C_2_ carbazoles, benzo[a]carbazole and benzo[c]carbazole).

We propose that fields more closely related in terms of filling sequence are expected to have closer similarity of their carbazole compositions. For a given exploration area, the carbazole compositional concentrations among different fields are expected to be different, due to the influence of numerous factors as described previously. Fields that are more closely related with respect to their filling sequence are expected to share more similar effects when compared to fields that are less related in terms of filling sequence. Consequently, the relative carbazole compositional concentrations, i.e. the proportion of the components, should be most similar among closely related fields than those among less related or unrelated fields. For example, although the carbazole compositional concentrations of sample D are very different from those of sample S (Table 1), the relative concentrations of these two samples are extremely similar (Table 2). The same is observed for W-H pair (Tables 1 and 2). Similarity in carbazole relative compositional concentrations between two reservoired oils can be measured by correlation of carbazole compositional concentrations. For example, Figs 5 and 6 show carbazole compositional correlations for oils from fields W, H, D and S. It can clearly be seen that the compositional correlation between fields W and H, and between D and S, is much closer than that between D and W. It is logical to infer that the W-H pair and D-S pair have closer filling relationships than the D-W pair. It is therefore proposed to employ carbazole compositional correlation coefficients to quantitatively measure carbazole compositional similarity, and thereby determine the filling relationships and migration pathways between the different fields.

The compositional correlation coefficients of all components (excluding C_0_ carbazole and benzo[c]carbazole, as previously discussed) between any two fields are evaluated and detailed in Table 3. The data show that compositional correlation coefficients of all components are very high and very similar among all the field pairs. As an example, Fig. 6a shows that the correlation of all components between fields D and S is only slightly higher than that between D and W. Since the compositional correlation coefficients of all components between any two fields are very similar, it is difficult to use them to quantify filling relationships among the fields.

Data points within the square insert (low-concentration components) in Fig. 6a indicate that the correlations of these data points for the two pairs could be very different. When these data points are plotted on a smaller scale (Fig. 6b), a clear and distinct difference between the correlations of the two pairs is observed. From the correlations it can be concluded that oil in field D is more closely related to the oil in field S than to field W. The low-concentration components are C_0_ carbazole, isomers of C_1_ and C_2_ carbazoles, benzo[a]carbazole and benzo[c]carbazole (Fig. 6b). Figure 6b illustrates that the correlation of these low-concentration components could be used to establish filling relationships between fields. Since C_0_ carbazole and benzo[c]carbazole are excluded from the compositional correlation analysis, the compositional correlation coefficients of the low-concentration components were calculated from the isomers of C_1_ and C_2_ carbazoles, and benzo[a]carbazole (Table 4). The results reveal large differences in the correlation coefficients of these low-concentration components among the combinations of different fields, ranging from 0.280 to 0.944. The different correlation coefficients are interpreted to represent different filling relationships among the fields.

**Figure 7:**
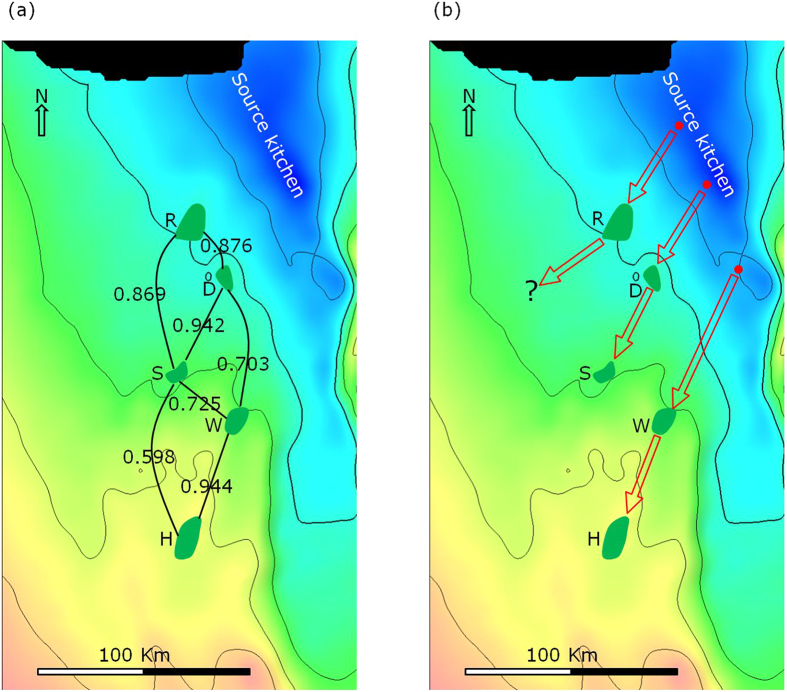
Geotracer compositional correlation coefficients (R^2^) between neighbouring fields (**a**) and deduced oil migration pathways (**b**).

The wide range in compositional correlation coefficients of the isomers of C_1_ and C_2_ carbazoles, and benzo[a]carbazole enables investigation of filling relationships among the fields, and thus oil migration pathways to be proposed. For convenience, the correlation coefficient of the isomers of C_1_ and C_2_ carbazoles, and benzo[a]carbazole is hereafter simply termed compositional correlation coefficient. To place the compositional correlation coefficients in context, values are marked on the Arab-A formation surface map for pairs of neighbouring fields (Fig. 7a). The correlation coefficients of the two pairs of neighbouring fields, D-S, and W-H, are extremely high (>0.94), significantly higher than that of other neighbouring fields.

**Figure 8:**
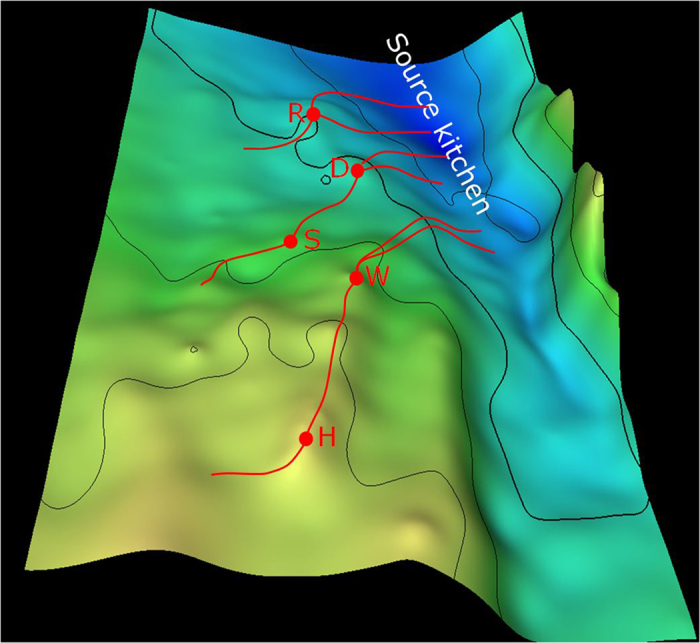
Location of the five oil fields on a 3D surface map of the Arab Formation of the Late Jurassic Epoch (source: Saudi Aramco) and the migration pathways (red lines) derived from a simple basin modeling analysis.

Filling relationships among the fields were investigated by examination of the fields one by one, starting with field D (Fig. 7a). In the analysis, an assumption was made that one spill point exists for a given trap, resulting in a single overflow pathway starting from the trap. A 3D surface map (Fig. 8) shows that, on the updip direction of field D, S and W are the two nearest traps. When trap D was filled to the spill point, the oil would potentially flow either to trap S or W. Since the correlation coefficient between D and S (0.942) is much higher than that between D and W (0.703), it can be concluded confidently that S is the successive trap to receive oil migrated from trap D.

This interpretation indicates that field W is not the recipient of the overflow from field D. The relative locations indicate that field W could receive its oil from the overflow of field S (Figs 7a and 8). If this is the case, since field S is much closer to W than W to H, the carbazole compositional variation, resulted from migration removal should be less between the former pair. Less compositional variation means higher compositional correlation coefficient. However, the compositional correlation coefficient for the S-W pair (0.725) is much lower than that for the W-H pair (0.944). Therefore, it is unlikely that trap W received its oil through S.

Similarly, the fact that the W-H compositional correlation coefficient (0.944) is much higher than that of the S-H pair (0.598) suggests that oil flowed from W to H, rather than from S to H. Note that the S-H compositional correlation coefficient is even lower than that between S and W (0.725), which we reasoned above that they do not fall along the same migration pathway.

A migration link between traps R and D or R and S is considered unlikely. If oil had flowed from R to D, the compositional correlation coefficient between R and D should be higher than or similar to that between D and S since the distance between the former pair is significantly shorter than between the latter pair (Fig. 7a). On the contrary, the correlation coefficient between R and D (0.876) is significantly less than that between D and S (0.942). It is, therefore, unlikely that oil flowed from R to D. The similar correlation coefficients between the R-S pair (0.869) and R-D pair (0.876) suggest that oil did not flow from R to S either. Furthermore, if oil had flowed to one of them from R, one of the correlation coefficients should be significantly higher than the other; this is not the case. To conclude, oil spilled from trap R flowed neither to D nor to S, but to other updip traps along a different migration pathway to the southwest (Fig. 7b).

In summary: 1) trap W did not receive its oil from either S or D; 2) D-S and W-H are two separate migration systems (Fig. 7b); and 3) any spill from trap R did not flow to trap D or S. Therefore, it is highly likely that structural or stratigraphic traps in the updip southwest direction of field R received oil spilled from R (Figs 7b and 8), and they represent good targets for further exploration.

Since trap D, as concluded, did not receive oil from a pathway passing through R, it must have received the oil from a different pathway. Similarly, trap W must have received its oil from a migration pathway different to those passing through fields R and D. In this scenario three migration pathways are required, each originating from a different part of the source kitchen, as illustrated in Fig. 7b. The D-S and the W-H pathways were determined with a high degree of confidence (Fig. 7b). Suggested pathways from the source kitchen to fields R, D and W (Fig. 7b) remain speculative, but account for the general filling directions and location of the source kitchen. The derived migration pathways (Fig. 7b) are totally different from the assumption of one migration pathway (Fig. 2).

**Figure 9:**
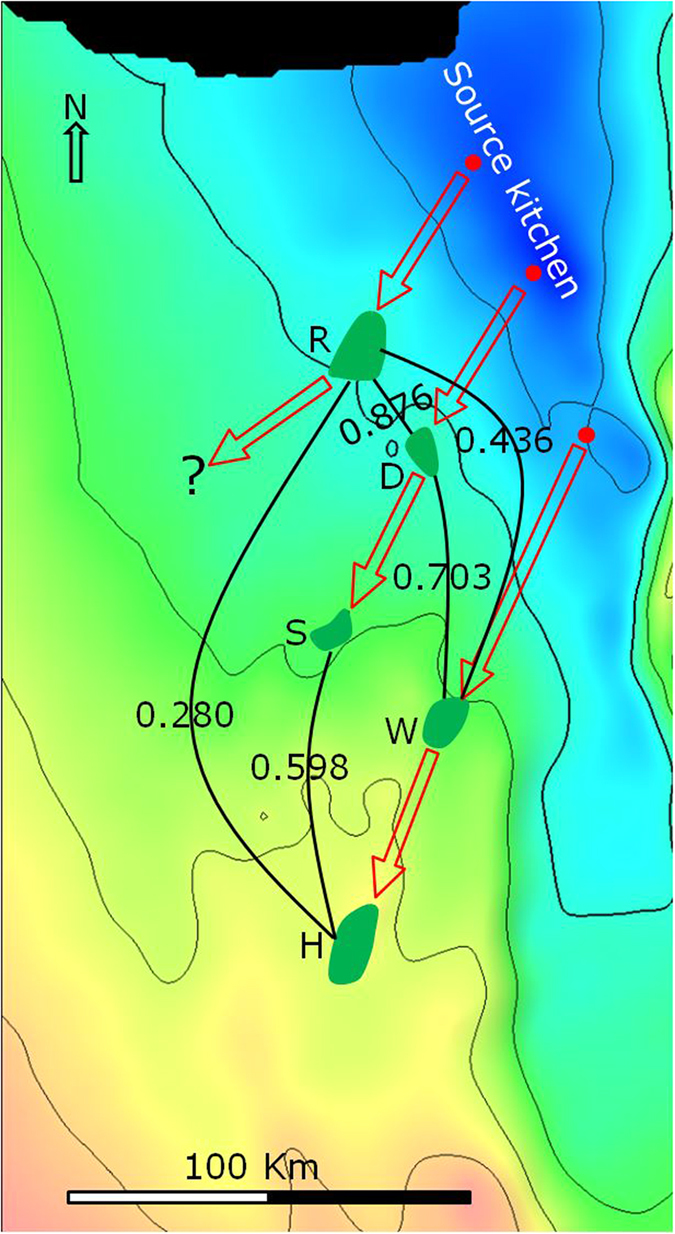
Carbazoles compositional correlation coefficients reflecting the influence of initial compositional concentration and removal from the oil during migration and accumulation.

Carbazole compositional concentration in reservoired oils is the final product of the initial concentration and subsequent effects of removal from the oil during migration and accumulation. Consequently, similarity of the compositional concentrations (represented by carbazole compositional correlation coefficients) between any two reservoired oils is a reflection of all these effects combined. As shown in Fig. 9, the large difference between the correlation coefficients determined for R–D (0.876) and R–W (0.436) pairs clearly indicates that the source of field R has a closer affinity to the source of field D, rather than to the source of field W. These observations suggest that the source of field R is geographically closer to that of field D than to the source of field W (Figs 8 and 9). Effects due to migration are observed when comparing the compositional correlation coefficients between D-W (0.703), and S-H (0.598) pairs (Fig. 9). The decrease in correlation coefficients from 0.703 to 0.598 is reflective of migration effects. That is, the difference in the relative carbazole concentrations is enlarged by migration and accumulation effects. This effect is also observed for R-W and R-H field pairs. Following migration (W to H), the correlation coefficient decreased from 0.436 to 0.280 (Fig. 9).

The absolute carbazole compositional concentrations between D and S are very different (Table 1). In consideration that D and S locate along the same migration pathway (Fig. 7b), the huge difference in their absolute carbazole compositional concentrations is attributed primarily to removal effects during migration and accumulation. In contrast, their relative carbazole compositional concentrations are very similar (Tables 2, 3, 4). The same observation is also found for W-H field pair. This indicates that although removal effects are considered the primary influence on the absolute carbazole compositional concentrations in these cases, the effect is much less significant on relative carbazole compositional concentrations. For the low-concentration components (Table 4), the compositional correlation coefficients between fields along the same migration pathways (D-S, and W-H, Fig. 7) are much higher than those between fields on different migration pathways (R-D, D-W, and R-W, Fig. 9). This indicates that, initial relative compositional concentration is the primary factor, while the removal effect is the secondary factor effecting the final relative compositional concentrations of the low-concentration components.

## Discussion

To check our results and conclusions, a simple basin modeling analysis has been conducted to reveal filling relationships between the five fields (Fig. 8). Comparing the migration pathways and filling relationships defined from the carbazole compositional correlation analysis (Fig. 7b) with those derived from the basin modeling analysis (Fig. 8) shows that conclusions derived from the two approaches are basically the same. That is, the conclusions derived from the carbazole compositional correlation analysis are supported by the basin modeling analysis, although these two approaches are totally different. Basin modelling is a process based approach, while the proposed approach is based on observation of the product, i.e., carbazole compositional concentrations in accumulated oils. The proposed approach provides an alternative, simple and quick method for assessing oil migration pathways.

**Figure 10:**
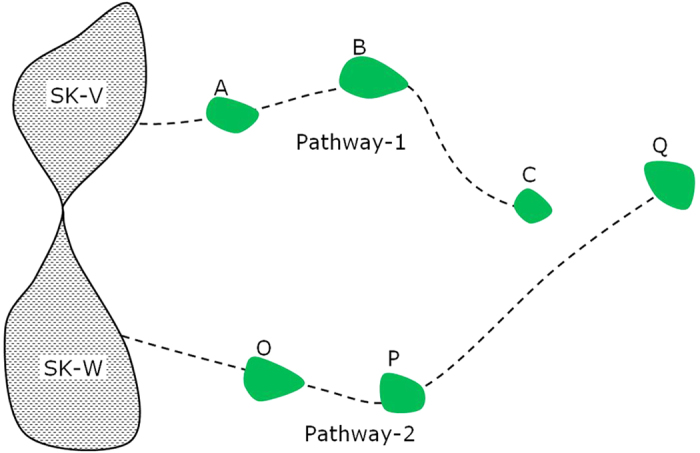
Illustrative example showing two migration pathways connecting two different source kitchens or different parts of the same source kitchen (SK-V and SK-W) with several oil fields.

The proposed theorem of the methodology can be summarised in an illustrative diagram (Fig. 10). SK-V and SK-W represent either two parts of the same source kitchen or two different source kitchens. A difference in the source and/or thermal maturity in SK-V and SK-W would result in different carbazole compositional concentrations in the generated oil. Oils generated from SK-V and SK-W kitchens travel along two migration pathways, accumulating in reservoirs A, B and C on Pathway-1, and O, P and Q on Pathway-2. During oil migration and accumulation, carbazole compositional concentrations are further altered along these two migration pathways. A spill-fill pair, such as A-B and O-P, would have a similar source input (initial relative compositional concentrations), and minimal difference in the removal effects, compared with non-spill-fill pairs, such as A-C along the same migration pathway, or B-P across Pathway-1 and Pathway-2. That is, a spill-fill pair should possess the most similar carbazole relative compositional concentrations, and so the highest correlation coefficients.

Pairs of spill-fill accumulations can, therefore, be determined by comparison of compositional correlation coefficients between neighbouring accumulations. Connection of the inferred spill-fill pairs allows migration pathways to be established.

Large variations in source type and thermal maturity between source kitchens SK-V and SK-W are expected to result in large differences in initial relative carbazole compositional concentrations. In such situations, correlations between pairs across Pathway-1 and Pathway-2, such as B-P and C-Q, will be low, thus making separation between the two pathways easier to establish. It is expected that the larger the effect of source type and thermal maturity, the greater the range of the carbazole compositional correlation coefficients among all field pairs. Therefore, effects due to source type and maturity variations would favor the use of the approach proposed herein. The five oils in the case study were inferred to have the same type source rock (Fig. 4) and similar maturity (Fig. 2). If their source types were not the same or the maturity difference was larger, it is expected that the compositional correlation coefficients would be more different and the determination of the migration pathways would be more apparent.

As previously stated, calculation of compositional correlation coefficients excluded C_0_ carbazole and benzo[c]carbazole, since the measured values for these components in field H were shown to be outliers (Table 1, Fig. 5b). The reason for the measured values of these two components in field H being outliers is not known. In future applications of the proposed method, there is no reason for the two components to be excluded.

The compositional correlation coefficients of all components between different fields are all very high and only slightly different (Table 3). The similarly high compositional correlation coefficients of all components are a result of the good correlation of high-concentration components (C_3_–C_6_ carbazoles and C_1_ and C_3_ benzocarbazoles, Table 5) and the large range in concentrations present in the samples (Figs 5 and 6). It is speculated that the low-concentration components (C_0_–C_2_ carbazole, benzo[a]carbazole and benzo[c]carbazole) are sensitive to subtle differences in source types, maturity, migration and accumulation, whilst the high concentration components are only sensitive to large differences in these factors.

Figures 5 and 6 show that for a pair of spill-fill oil accumulations (W-H, D-S), the compositional concentrations in one accumulation are nearly linearly proportional to those of the other. The removal of carbazole components from the oil during migration and accumulation are reportedly due to adsorption to the solid surface of the rock matrix^11^. The observed linear relationship in the compositional concentrations for a spill-fill pair can only result from two combined conditions: 1) carbazole adsorption in a migration system is geologically an instant process, i.e. behaves as an equilibrium adsorption, which has been previously demonstrated by laboratory experiments^11^; and 2) the Freundlich adsorption equation^27^ with the constant parameter n = 1 applies, and that the adsorption constant (K) in the Freundlich equation is similar for all carbazole components.

**Figure 11:**
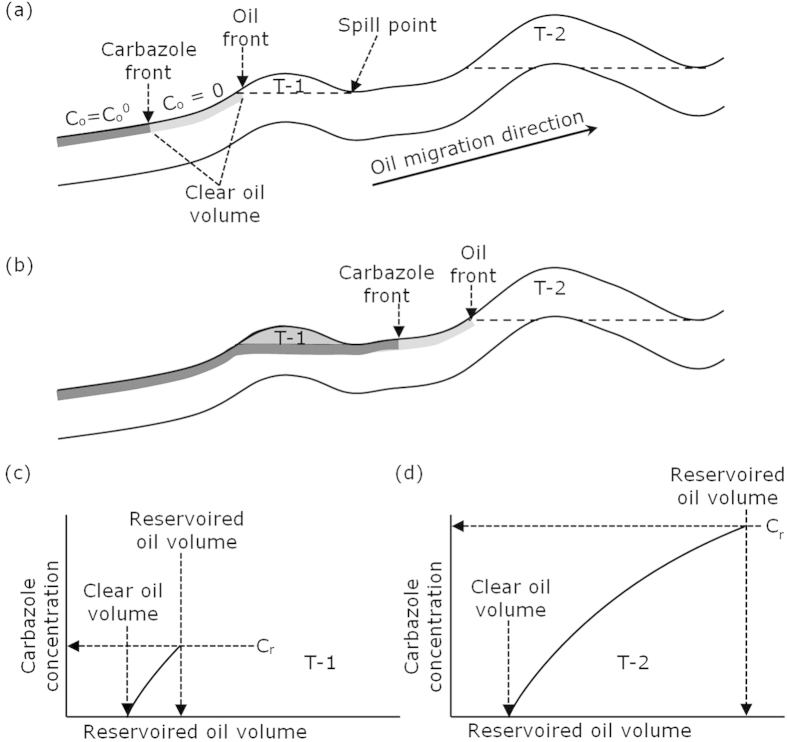
Diagram showing carbazole distribution along a migration pathway (**a**,**b**), and in reservoired oil accumulations (**c**,**d**). Note the positive effect of the volume of the reservoired oil on carbazole concentration (**c**,**d**). C_r_ is the carbazole concentration of a reservoired oil, C_o_ concentration in migrating oil, C_o_^0^ initial concentration out of the source. T-1 and T-2 are two successive traps along the migration pathway (Modified from Yang *et al.*, 2005)^11^.

Although field S is the successor of field D on the deduced D-S migration pathway (Fig. 7b), the carbazole concentration of field S is over three times higher than that of field D (Table 1). This observation appears to be counterintuitive. Actually, the resulted concentration of carbazole in an oil accumulation is not entirely due to migration distance. Rather, it is a complicated function of multiple factors, including inherent properties of the migration systems, chemical properties of carbazoles, and volume of the reservoired oil^11^. As illustrated in the diagrams in Fig. 11, mathematical modelling shows that, due to its equilibrium adsorption nature in geological time scale, carbazole concentration behind a carbazole front is constant and the same as the initial concentration expelled from the source (C_o_^0^), and zero in front of the carbazole front^11^. The distance between the carbazole front and the oil migration front increases with oil migration distance. A trap initially receives oil that contains no carbazoles, i.e. clear oil before the carbazole front, then oil with the initial carbazole concentration (C_o_^0^) (Fig. 11a,c). In reservoir, the carbazoles are further removed by adsorption to the solid matrix of the reservoir rock, and homogenise within the accumulated oil. Carbazoles are continuously brought into the reservoir by the migrating oil, and their concentration increases until the trap is fully filled (Fig. 11c). Obviously, the larger the reservoired oil volume, the higher the carbazole concentration. When a trap is fully filled, oil begins to migrate further along the migration pathway. At this moment the carbazole front is in the same position as the oil front, i.e. at the spill point (Fig. 11a). During the following stage of oil migration, the carbazole front gradually lags behind the oil front (Fig. 11b). This process is repeated for subsequent traps, which accumulate clear oil first, followed by oil containing the initial carbazole concentration (Fig. 11b,d). Mathematical modelling demonstrates that carbazole concentration in reservoired oils is negatively correlated to the relative migration distance (i.e. the distance the oil has migrated from the source or the preceding trap to reach the current trap), but positively correlated to the volume of reservoired oil^11^.

The relative migration distance for oil in field D (from the source to trap D) is similar to that for oil in field S (from D to S), approximately 40 km (Fig. 7b). The volume of oil in trap S is double that found in trap D. Since traps D and S have similar relative migration distances, but the volume of oil in trap S is much greater than that in trap D, according the mathematical model result^11^, carbazole concentrations in field S are far higher than that in field D. This case is similar to the example scenario illustrated in Fig. 11, in which fields D and S are similar to trap T-1 and T-2, respectively.

In the above discussion it has been assumed that when a trap, such as T-1 in Fig. 11a, becomes fully filled, the later oil will flow beneath the trap and exit at the spill point (bypassing mechanism). In a different scenario, the later oil may replace the earlier oil in the trap, which, in turn, migrates updip to fill another trap (replacing mechanism). If the replacing mechanism was true, the concentration of geotracers observed in the updip trap would have to be much lower than that found in the downdip trap due to repeated removal of geotracers during the longer migration further updip. In the case of fields D and S, the concentration of geotracers in the updip trap S is over three times higher than that found in the downdip trap D (Table 1). The D-S example agree with a bypassing, rather than a replacing, mechanism. In other words, when trap D was fully filled, later oil charge moved beneath or around the trapped oil and continued its migration toward trap S.

Direct correlations of geotracer concentrations to oil migration distance are dependent on numerous factors, including source rock type and maturity^2^,^12^,^15^,^16^,^17^,^18^,^19^,^20^,^21^,^22^,^23^, migration channel dimension, adsorption capacity of rock matrix and volume of oil in place^11^. In reality, not all these factors can be readily established for a given study area. Using the proposed method, a prior knowledge of the above factors is not a prerequisite for straightforward mathematical manipulation. This new method provides an alternative, simple, and practical approach for assessing petroleum migration pathways in the search for new discoveries.

## Methods

Carbazole compounds were separated by solid phase extraction (SPE) using C18 non-end-capped cartridge (ISOLUTE 220-0050-B, 500 mg C18, 3 mL)^2^. Ca. 100 mg of each oil sample was dissolved in 1 mL n-hexane and the solution sonicated for 10 minutes. SPE cartridges were equilibrated using 2 mL n-hexane. Following equilibration sample solutions were transferred to SPE cartridges. Fraction 1 was eluted using 5 mL n-hexane and fraction 2 (comprising the target carbazole compounds) was eluted with successive volumes of dichloromethane (5 mL). Fraction 2 was reduced to 1.5 mL under N_2_, from which 500 μL was transferred to a 2 mL glass vial. 10 μμL of internal standard, 9-phenylcarbazole solution (14.6 ppm) in dichloromethane was added to each carbazole fraction for quantitation. 1 μL of this aliquot (sample plus internal standard) was used for GC-MS analysis. An Agilent 6890N GC interfaced with Agilent 5973 inert quadruple MS was used for the analysis of carbazoles. The GC was equipped with a split/splitless injection system. The split ratio employed was 10:1. A DB-1 capillary column (60 m × 0.25 mm × 0.25 μm) was used. Helium was used as a carrier gas at a constant flow of 1.9 mL/min. The GC oven temperature was programmed as follows: 60 °C (2 min), then ramped to 180 °C at 20 °C min-1, held for 1 min, increased to 310 °C at 4 °C min-1 and held at the final temperature for 10 min. Using an electron energy of 70 eV and a source temperature of 230 °C the MS was operated in SIM mode. The C_0_ benzocarbazoles were monitored using the m/z 217 fragmentogram, while C_1_ and C_3_ benzocarbazoles were monitored using their diagnostic m/z 231 and 259 fragmentograms, respectively. Carbazoles were monitored using: C_0_ carbazole (m/z 167), C_1_ carbazoles (m/z 181), C_2_ carbazoles (m/z 195), C_3_ carbazoles (m/z 209), C_4_ carbazoles (m/z 223), C_5_ carbazoles (m/z 237) and C_6_ carbazoles (m/z 251). The internal standard 9-phenylcarbazole was monitored using its m/z 243 chromatogram. The retention times of benzocarbazole isomers (a, b, and c) were also verified against authenticated standard compounds obtained from Chiron, while alkylated carbazoles were identified based on comparison with the literature^28^.

Oil charge and migration pathways were evaluated by following two simple steps. Firstly, compositional correlation coefficients of low-concentration carbazole components for each pair of fields for the exploration area were calculated. Secondly, compositional correlation coefficient of each pair of neighbouring fields was compared with those of adjacent pairs to check if a filling link can be established. It is recommended to start with the easiest pairs first. This way, conclusions derived previously can be used in subsequent analysis of more complex scenarios.

## Additional Information

**How to cite this article**: Yang, Y. and Arouri, K. A Simple Geotracer Compositional Correlation Analysis Reveals Oil Charge and Migration Pathways. *Sci. Rep.* **6**, 23066; doi: 10.1038/srep23066 (2016).

## Figures and Tables

**Figure 1:**
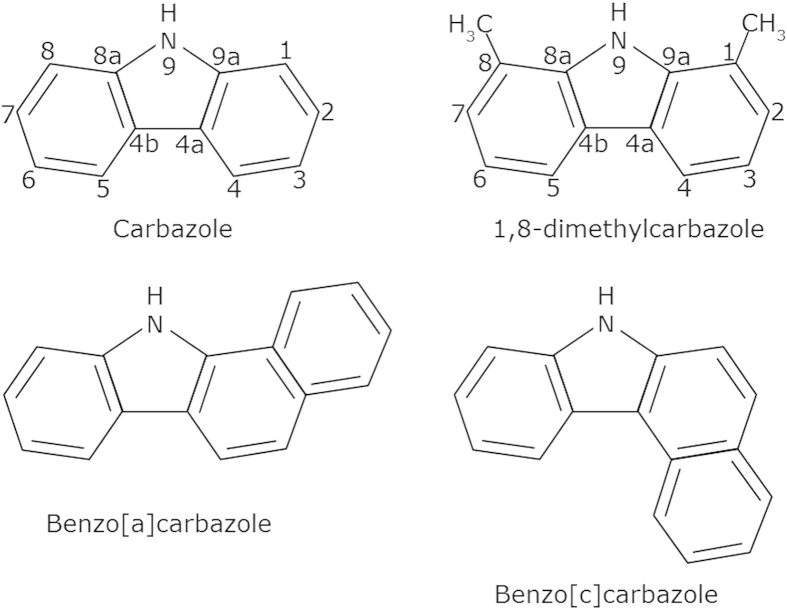
Chemical structure of some representative carbazole components.

**Table 1:**
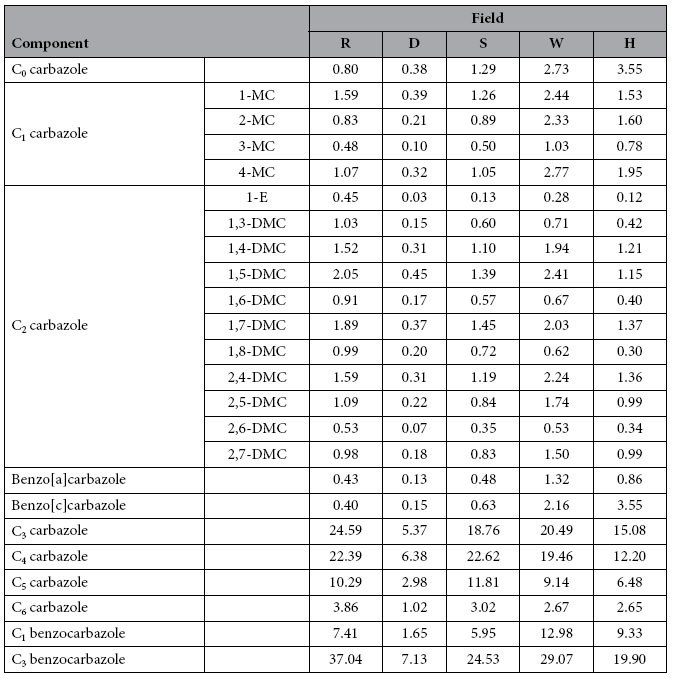
Carbazole compositional concentrations (μg/g).

MC: methyl carbazoles; DMC: dimethyl carbazoles; E: ethyl carbazole.

**Table 2:**
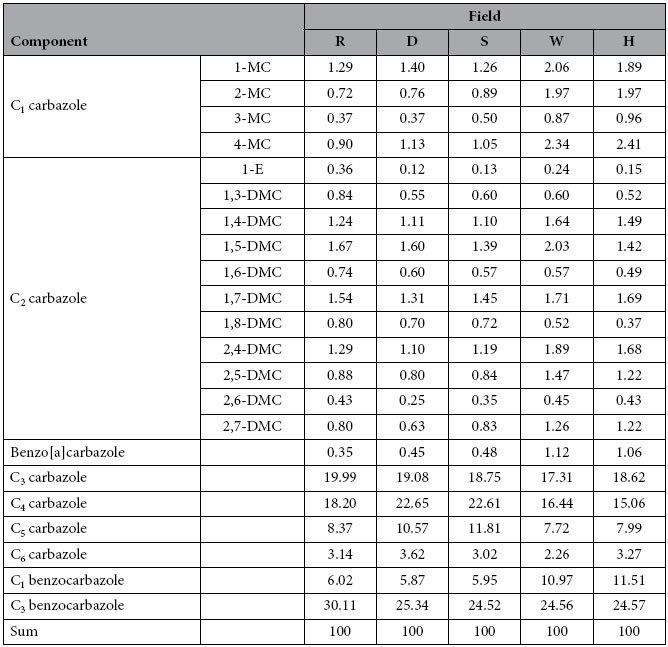
Carbazole relative compositional concentrations (%).

**Table 3:** Compositional correlation coefficients (R^2^) of all components (excluding C_0_ carbazole and benzo[c]carbazole).

	R	D	S	W	H
R		0.963	0.951	0.963	0.955
D	0.963		0.998	0.942	0.924
S	0.951	0.998		0.934	0.915
W	0.963	0.942	0.934		0.996
H	0.955	0.924	0.915	0.996	

**Table 4:** Compositional correlation coefficients (R^2^) of low-concentration components (isomers of C_1_, C_2_ carbazoles and benzo[a]carbazole).

	R	D	S	W	H
R		0.876	0.869	0.436	0.280
D	0.876		0.942	0.703	0.536
S	0.869	0.942		0.725	0.598
W	0.436	0.703	0.725		0.944
H	0.280	0.536	0.598	0.944	

**Table 5:** Compositional correlation coefficients (R^2^) of high concentration components (C_3_–C_6_ carbazoles and C_1_, C_3_ benzocarbazole).

	R	D	S	W	H
R		0.913	0.883	0.924	0.915
D	0.913		0.994	0.838	0.782
S	0.883	0.994		0.815	0.755
W	0.924	0.838	0.815		0.986
H	0.915	0.782	0.755	0.986	
